# A reproducible benchmark of QRS detection algorithms across diverse ECG datasets and noise conditions

**DOI:** 10.1038/s41598-026-53724-9

**Published:** 2026-05-20

**Authors:** Simon Maximilian Wolf, Tim Rahlmeier, Stefan Lustfeld, Detlef Schoder

**Affiliations:** Cologne Institute for Information Systems, University of Cologne, Universitätsstraße 24, 50931 Cologne, Germany

**Keywords:** R-peak detection, Benchmarking, Noise resilience, ECG, Computer science, Diagnostic markers, Electrodiagnosis

## Abstract

Accurate R-peak detection in electrocardiograms is critical for heart rate monitoring, heart rate variability analysis, and cardiac condition diagnosis. However, reliable detection remains challenging in real-world scenarios due to noise, artifacts, and signal variability. A key limitation in current research is the lack of reproducibility and comparability, as algorithms are often tested on varying datasets, hindering direct performance comparisons. To address this, we benchmark 17 R-peak detection algorithms, encompassing traditional signal processing, machine learning, and deep learning approaches, within a unified evaluation framework using five open-access ECG datasets from the PhysioNet platform. These databases represent diverse conditions, including long-term monitoring, arrhythmias, and noisy environments, enabling a standardized evaluation. Our results reveal that under a strict cross-dataset generalization setting, in which ML and DL models were trained on a single dataset without any target-domain adaptation, traditional signal processing methods provided more consistent overall performance. This highlights a trade-off between peak performance on familiar data and generalizable performance under distribution shift, whose extent for data-driven methods may depend substantially on training diversity. To support reproducibility and future benchmarking, we provide a fully open evaluation framework including all implementations, dataset references, and evaluation pipelines. These findings offer guidance for researchers and clinicians selecting R-peak detection algorithms for diverse clinical and practical scenarios.

## Introduction

R-peak detection focuses on identifying the most prominent peak within the QRS complex of an electrocardiogram (ECG), known as the R-wave, which reflects the moment of greatest electrical activity during the cardiac cycle^1^. Accurate detection of R-peaks is fundamental for calculating heart rate (HR) and heart rate variability (HRV), metrics that are widely used in clinical and research settings^2^. HRV analysis, in particular, is critical not only for diagnosing cardiovascular diseases and monitoring cardiac health but also for investigating broader physiological and pathological states, including stress, epilepsy, and other neurological disorders^3–5^. The centrality of these metrics in medical and scientific practice underscores the importance of accurate and reliable R-peak detection. While the term R-peak detection is commonly used in the context of HR and HRV analysis, most algorithms fundamentally aim to detect the QRS complex. In this study, we follow the convention of referring to R-peak detection, as the R-peak serves as the fiducial point for evaluation. Nevertheless, the underlying task corresponds to QRS complex detection.

**Fig. 1:**
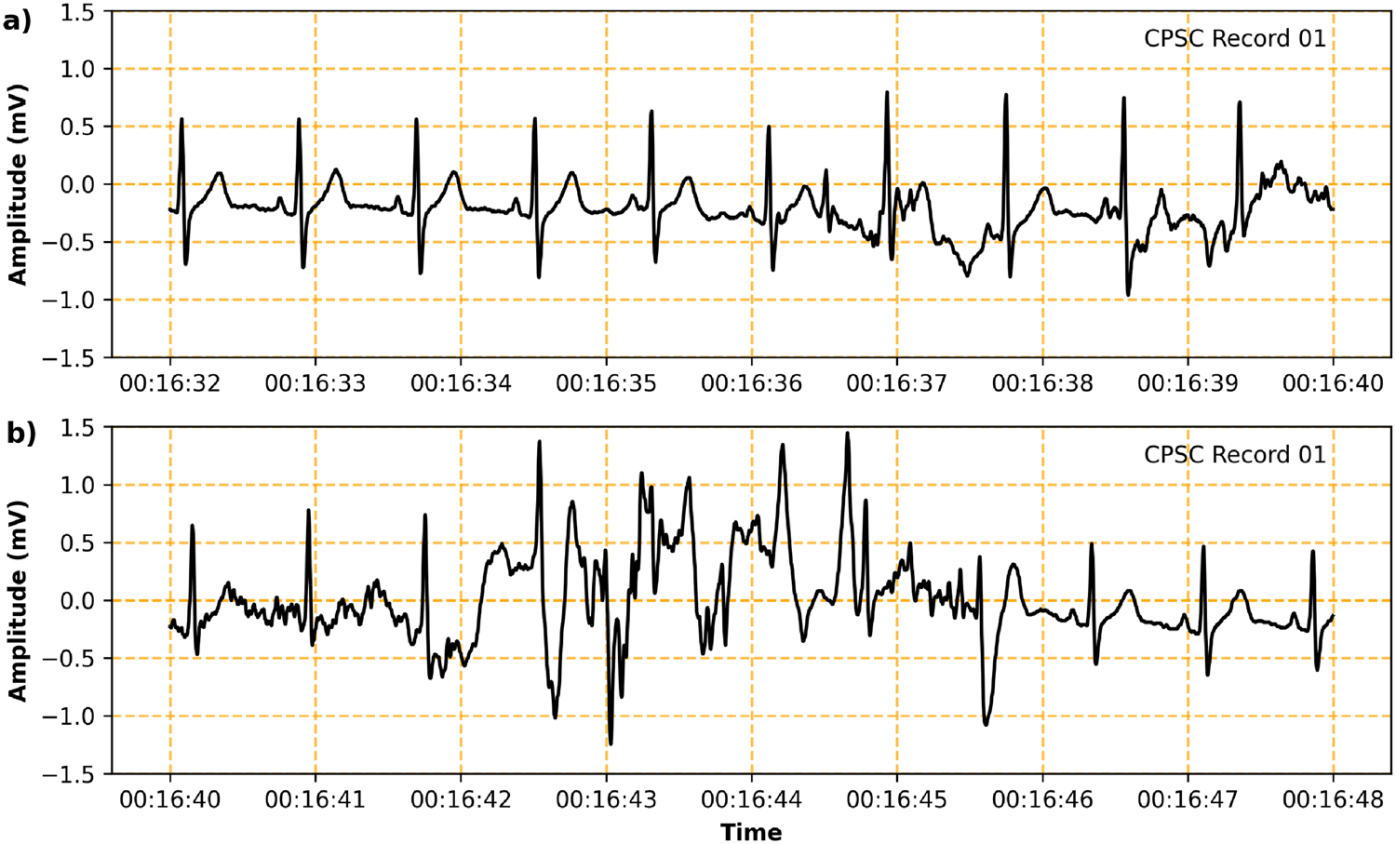
R-peak detection—An easy task? The figure illustrates two consecutive segments of the same ECG recording captured by a wearable device. The presence of motion artifacts in (**b**) makes it challenging to accurately detect R-peaks, whereas (**a**) shows a clean, interference-free signal, highlighting the complexity of this task.

Detecting R-peaks, however, involves numerous challenges. ECG signals are susceptible to interference from various sources, including motion artifacts, baseline wander, and electromagnetic noise, all of which can obscure the QRS complex^5^. Moreover, the morphology of ECG signals exhibits significant heterogeneity across patients, influenced by factors such as age, cardiac anatomy, and pathological conditions that limit generalizability particularly for deep learning (DL) models^6,7^. These variations are especially pronounced in patients with arrhythmias or other atypical ECG patterns, where reliable R-peak detection is critical for accurate diagnosis and monitoring^8^. Furthermore, errors in R-peak detection can have cascading consequences, potentially undermining downstream analyses. As Jeppesen emphasizes, “Even a single missed or wrongly detected peak can have great influence on all of the HRV analysis”^9^. However, the development of robust and generalizable R-peak detection methods, capable of performing reliably across diverse patients and effectively handling variations such as noise, motion artifacts, and other disturbances, remains a key challenge in the field^6,10,11 (Fig. 1)^.

Since the introduction of the widely adopted algorithm by Pan and Tompkins^1^, which became one of the most influential early approaches to R-peak detection, significant progress has been made in this field. Subfields have emerged that focus on optimizing algorithms for near real-time processing and minimizing memory usage for resource-constrained systems^10^. Concurrently, efforts have aimed to develop innovative approaches that achieve maximum accuracy, including advanced preprocessing techniques to improve traditional signal-processing methods and the integration of machine learning (ML) and DL^10^. These advancements have enabled reported accuracies of up to 99%, depending on dataset characteristics and patient-specific conditions^10,11^. However, recent reviews, such as that by Dogan and Dogan^11^, highlight a persistent need for noise-robust methods that perform consistently on real-world, heterogeneous, and noisy data. Despite this progress, the field still faces challenges regarding the comparability and reproducibility of results: studies often fail to share their code, use inconsistent evaluation datasets, define true positives and metrics differently, and handle poor-quality recordings in non-standardized ways^11–13^. This fragmentation hampers fair and transparent performance comparisons, making it difficult to assess whether a method performs reliably across diverse conditions. As a result, it remains unclear whether a method generalizes well or if its performance is limited to specific scenarios, for example, handling strong motion artifacts effectively while failing under other conditions, such as electromagnetic interference. Yet, this is precisely the question that matters most in practice: when an algorithm must be selected out of the box, without prior knowledge of patient characteristics, without dataset-specific training or fine-tuning, and without risk of data leakage, which approaches actually generalize well? Throughout this paper, we use the term *generalizability* in this specific, practical sense: an algorithm’s ability to perform reliably on unseen data without any form of adaptation to the target domain. To the best of the authors’ knowledge, no existing work has systematically addressed this question through a unified, transparent, and reproducible benchmark that the research community can adopt and extend.

In this study, we address this gap by conducting a systematic benchmarking of 17 re-implemented R-peak detection algorithms, encompassing traditional signal processing methods, ML approaches, and state-of-the-art DL techniques. To ensure comparability, we evaluated all algorithms on the same five open-access ECG datasets from the PhysioNet^14^ platform. These datasets focus on different dimensions, such as long-time monitoring^15^, arrhythmias^16^, noisy environments^17^, resting^18^ and motion-related variations^19^, providing a comprehensive and realistic basis for evaluation. To ensure reproducibility, we prioritized algorithms with detailed documentation and available source code. However, when no source code was available, we re-implemented methods based on published descriptions to incorporate them into our analysis. By standardizing datasets and defining clear evaluation criteria such as a unified definition of true positives, this study aims to provide a transparent and objective assessment of algorithm performance under diverse conditions.

Our results offer key insights into the trade-offs between algorithm complexity, accuracy, and robustness. Under the evaluated out-of-the-box setting, in which all ML and DL models were trained on a single dataset and applied without adaptation to unseen databases, traditional signal processing methods demonstrated greater consistency across diverse and heterogeneous datasets. ML and DL approaches, while achieving strong performance on specific datasets and superior robustness under high-noise conditions, showed greater sensitivity to distribution shift from their training source. This likely reflects the constraints of the adopted training strategy rather than an inherent ceiling of data-driven methods, as more diverse training data could be expected to improve their cross-dataset transferability. A persistent trade-off between peak performance on specialized data and consistent generalization across diverse conditions remains evident regardless of paradigm.

Beyond benchmarking, our findings highlight broader implications for the field. First, under the evaluated training conditions, they reveal sensitivity to distribution shift in several ML-based approaches, highlighting the need to improve noise resilience and the breadth of training data for better generalizability of these methods. Second, they provide guidance for researchers and clinicians seeking to select appropriate algorithms for specific clinical or research applications. Finally, this study underscores the importance of reproducibility and standardization in R-peak detection research. To this end, we provide not only our results but also a fully open and reproducible evaluation framework, including all re-implementations, datasets, and evaluation pipelines, intended to serve as a foundation that future studies can build upon, whether to benchmark novel algorithms against established baselines or to challenge and refine the methodology itself.

## Theoretical background

### R-peak—cause and identification

**Fig. 2:**
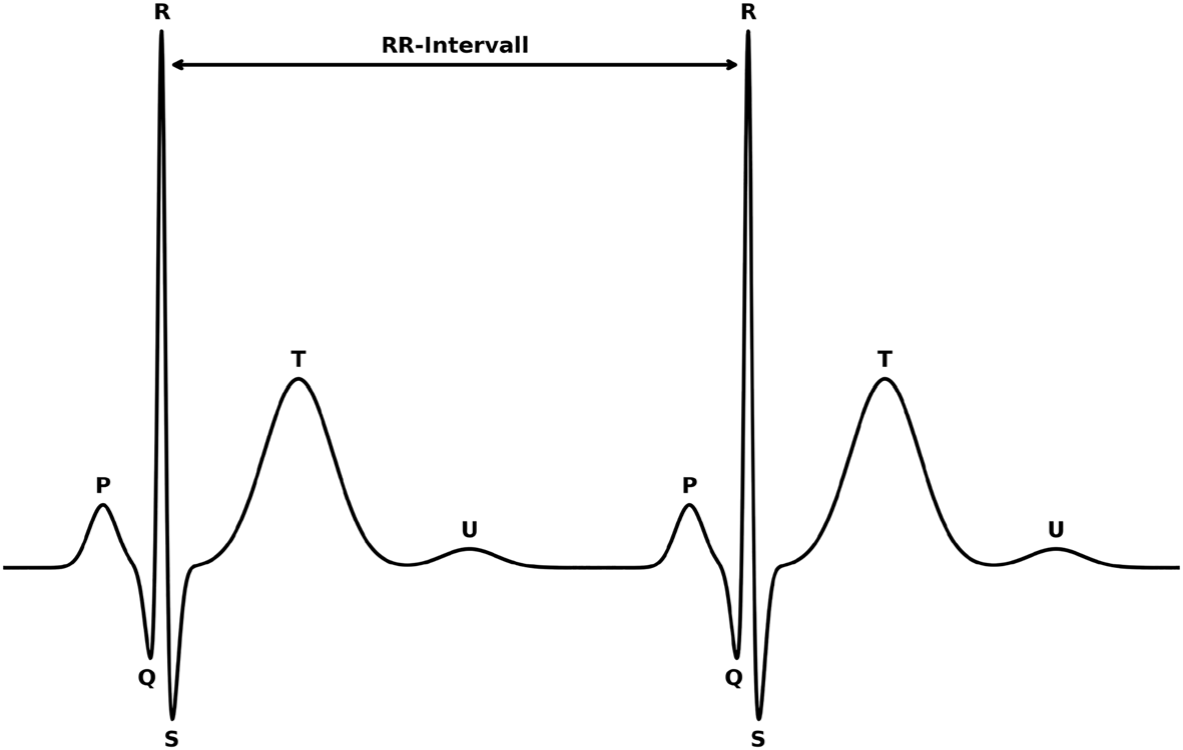
Schematic heart cycle with labeled P-, Q-, R-, S-, T- and U-wave.

The ECG is commonly used to assess heart rhythm, monitor heart rate, and identify potential abnormalities in cardiac function^20^. Electrodes placed on the skin detect voltage changes caused by heart muscle contractions, measuring the heart’s activity^21^. These changes are displayed as a waveform, typically consisting of P-, QRS-, and T-waves, each corresponding to different phases of the cardiac cycle (Fig. 2).

In a healthy heart, the P-wave marks atrial contraction^22^, followed by the QRS complex (Q-, R-, and S-waves), which represents ventricular depolarization and is the most significant component for R-peak detection^23^. The T-wave indicates ventricular repolarization^16^, while the U-wave is a minor deflection that is often barely visible or absent altogether^24,25^, with some studies omitting it entirely^26^. Although the P- and T-waves are diagnostically important, absent P-waves may indicate atrial fibrillation^22^, abnormal T-waves can suggest myocardial ischemia^16^, in the context of R-peak detection, they are primarily treated as interference that should be minimized^27^. Abnormalities in the slope, width, amplitude, and timing of the QRS complex can indicate various cardiac diseases^21,24^, and localizing the QRS complex also aids in identifying the P- and T-waves^28^.

The R-peak is the highest point in the R-wave. It is crucial because it represents the most distinct and easily identifiable point in the cardiac cycle, making it essential for accurate calculations of the R-R interval, which is used to measure heart rate and heart rate variability. Focusing on the R-peak, rather than the broader R-wave, is important because the peak provides a precise reference point for timing, ensuring consistency and accuracy in interval measurements. However, detecting the R-peak can be challenging due to noise and artifacts in the signal, leading to potential misidentification^29^.

**Fig. 3:**
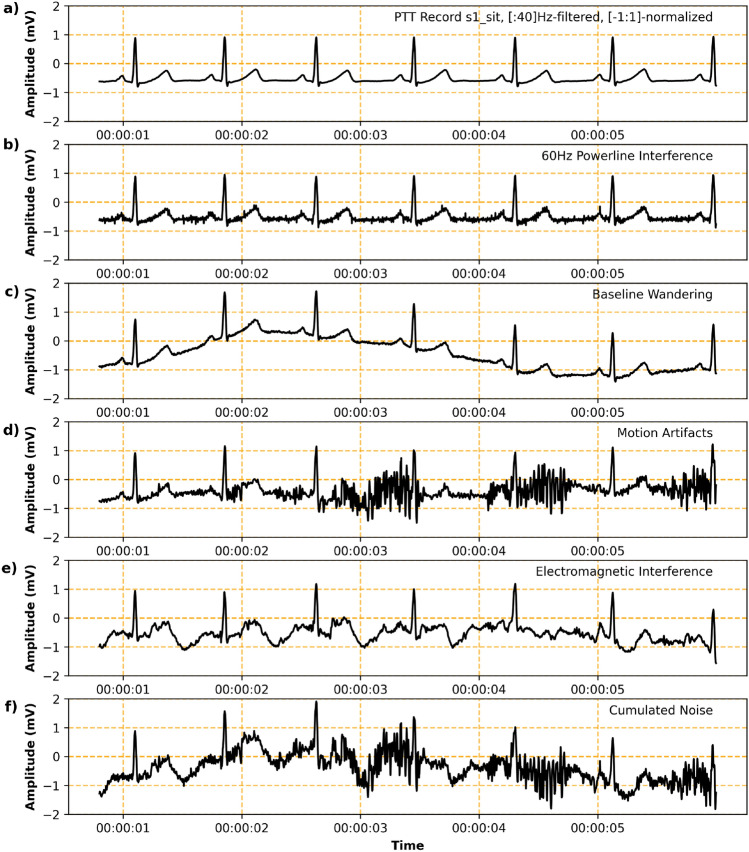
Comparison of ECG signal distortions under various noise conditions. Panel (**a**) shows the clean ECG signal of recording s1_sit from the Pulse Transit Time Database, normalized to a range of [-1, 1] and filtered to exclude frequencies above 40 Hz. Panels (**b**) through (**f**) illustrate the impact of different noise sources: (**b**) 60 Hz powerline interference, (**c**) baseline wandering, (**d**) motion artifacts, (**e**) electromagnetic interference, and (**f**) cumulated noise. The noise is added using the noise files from the MIT Noise Stress Test Database.

Noise in ECG signals can arise from various sources that impair signal quality (Fig. 3). Powerline interference occurs due to electromagnetic disturbances from power lines, typically manifesting at a frequency of 50/60 Hz in the signal^30^. Baseline wandering is often caused by breathing movements or slow shifts in electrode positioning, resulting in a drifting baseline of the ECG signal^21,31^. Motion artifacts stem from patient movements, causing mechanical disturbances between the electrodes and the skin^24,32^. Electromagnetic interference can also be introduced by external electronic devices near the ECG system, further distorting the signal. These disturbances complicate the accurate detection of R-peaks and other key waves in the ECG^22,33^. In practice, multiple noise sources often occur simultaneously, compounding the challenge^22^.

### Common signal preprocessing steps

Noise poses the primary challenge in R-peak detection, and different algorithms handle it with varying degrees of success. As a result, various ECG signal processing techniques are applied to enhance the clarity of the QRS complex or the R-peak before detection^31^. These processing techniques are widely used across disciplines, but they are particularly crucial for R-peak detection methods and can be quite extensive before the processed signal is passed to the detection algorithms. Each algorithm presented later employs some form of signal processing, making preprocessing and the detection algorithm a combined package. Key concepts are briefly introduced here to provide a better understanding of the detection methods discussed later.

Taking the absolute value and squaring the signal are two common techniques used to improve R-peak detection. The absolute value converts negative R-peaks into positive values, simplifying the detection process. Squaring the signal amplifies higher values, such as R-peaks, while minimizing smaller values like P- and T-waves^1,34^.

First- and second-order differentiation are also crucial for R-peak detection. First-order differentiation captures changes in the slope of the signal, making it particularly useful for identifying the characteristic steep positive and negative slopes of the QRS complex^1,34^. Second-order differentiation goes further by describing the rate of change of the first derivative, providing additional insights into the signal’s structure^32^.

Various filtering techniques are applied to refine the signal. A low-pass filter removes high-frequency noise, such as powerline interference, allowing only relevant low-frequency ECG components to pass through. In contrast, a high-pass filter eliminates low-frequency disturbances, like baseline wandering, by preserving higher frequencies. A bandpass filter combines both approaches, allowing only a specific frequency range, typically between 5 and 15 Hz, to pass through, which is commonly used for ECG signal preprocessing^11^.

Another technique used is the moving average, which smooths the signal by averaging each point with its neighboring points. This reduces high-frequency fluctuations and irrelevant peaks, decreasing the computational load for R-peak detection algorithms. Similarly, the peak envelope technique defines a line connecting all relevant positive or negative peaks, reducing the number of irrelevant peaks in the signal^35^.

Fourier transformation decomposes the signal into its frequency components, enabling targeted amplification or suppression of specific frequencies. This is particular used for emphasizing the QRS complex while attenuating disruptive waves like the T- and P-waves. The short-time Fourier transformation further enhances the analysis by dividing the signal into short time segments, making it more effective for analyzing non-stationary signals like ECGs^30,36^.

The wavelet transformation provides both time and frequency information, making it suitable for detecting QRS complexes in non-stationary signals. By using mother wavelets, which analyze the signal at different scales and positions, both short- and long-term frequency variations can be captured effectively^26^.

In addition to these common techniques, there are other less widely used methods. Shannon energy emphasizes high-energy regions, such as R-peaks, while suppressing noise^28^. Empirical mode decomposition breaks the signal into multiple intrinsic mode functions, each representing the envelopes of positive and negative peaks^35^. Finally, the Hilbert transformation converts the signal into an analytic form, making it particularly useful for highlighting transitions, such as R-peaks, in noisy environments^28^.

### Machine learning and deep learning approaches

In addition to traditional signal processing, ML and DL methods have been increasingly applied to R-peak detection. Classical ML approaches, such as K-means clustering, have been used to classify signal segments based on extracted features^25^. More recently, DL architectures have gained prominence due to their ability to learn discriminative features directly from raw or minimally preprocessed signals. Among the most commonly employed architectures are one-dimensional convolutional neural networks (1D-CNNs), which apply learned filters to capture local morphological patterns such as the steep slopes and high amplitude characteristic of the QRS complex^26,37,38^. Recurrent neural networks (RNNs), particularly long short-term memory (LSTM) networks, model temporal dependencies across consecutive heartbeats, which can help distinguish true R-peaks from noise by leveraging the rhythmic context of surrounding beats^39,40^. Some approaches combine both paradigms, using CNNs for local feature extraction followed by RNNs for sequential context^40^. However, these data-driven approaches typically require large amounts of annotated training data and may struggle to generalize when the characteristics of unseen recordings differ substantially from the training distribution^6^. A comprehensive review of these and further approaches can be found in Dogan and Dogan^11^. In this study, we include representative algorithms from each of these categories to ensure methodological breadth in our benchmark, rather than aiming for an exhaustive survey of all proposed architectures.

## Method

Given the diversity of available approaches and the persistent lack of standardized comparisons across methodological paradigms, a unified benchmarking framework is needed. The following section describes how we selected databases and algorithms to enable such a systematic and reproducible evaluation.

### Selection of databases

The performance of R-peak detection algorithms is often assessed using recordings from specific ECG databases, which include R-peak annotations provided either by medical experts or generated automatically by other algorithms. However, the number and type of databases used in these evaluations can vary significantly across studies^11^. For instance, among our selected R-peak detection algorithms, the number of databases used for performance evaluation ranged from 1 to 11. In some cases, authors selectively excluded certain recordings or segments of recordings known to challenge their algorithm, potentially inflating their reported detection performance^32^.

PhysioNet^14^, a widely utilized platform for medical databases across various applications, hosts over 300 datasets as of September 2024, with more than 200 available as open access. Its prominence in the field extends to ECG datasets, making it an ideal resource for the systematic selection of evaluation databases in this study.

To ensure reproducibility and facilitate future benchmarking of R-peak detection algorithms, we restricted our selection to open-access databases. After screening, we identified 37 datasets containing ECG leads with annotated R-peaks. Of these, 13 datasets had annotated R-Peaks which were either set or confirmed exclusively by medical experts, providing a higher level of reliability.

From this subset, five databases (MIT Long Term^15^, Fantasia^18^, Pulse Transit Time^19^, MIT Arrhythmia^16^ & MIT Noise Stress Test^17^) were selected for their broad coverage of relevant conditions, including long-term recordings, data from both resting and dynamic states, arrhythmic episodes, and controlled levels of noise. The remaining eight datasets were deemed unsuitable due to redundancy, extreme specificity (e.g., fetal ECG or data collected during magnetic resonance imaging procedures), or insufficient recording duration (e.g., recordings lasting only 10 seconds). Additionally, two databases were excluded as their R-peak annotations were reported to contain errors.

**Table 1:** Overview of the databases. To rule out data leakage, the CPSC database is the one used for training, while the others are used for evaluation.

	Total length (hh:mm:ss)	R-peaks (amount)	Sample rate (Hz)	Recordings (amount)	Recording length (hh:mm:ss)		
					Minimum	Average	Maximum
CPSC	244:08:44	1026095	400	10	22:49:45	24:24:53	25:53:09
MIT Long Term	147:27:27	668735	128	7	13:56:56	21:03:47	23:52:52
Fantasia	79:46:23	282583	250	40	01:43:54	01:59:42	02:36:10
Pulse Transit Time	09:00:34	46405	500	66	00:08:04	00:08:08	00:09:09
MIT Arrhythmia	24:04:27	109494	360	48	00:30:06	00:30:06	00:30:06
MIT Noise Stress Test	06:01:07	25590	360	12	00:30:06	00:30:06	00:30:06

In addition to the five evaluation databases, we included the publicly available China Signal Processing Challenge (CPSC) database^41^, as a neutral dataset for the initial training of the selected ML algorithms (Table 1). The CPSC dataset was split into 90% training and 10% test data using a random split at the recording level. The test partition served as a development set for monitoring training progress and preventing overfitting. Once training was completed, all model parameters were frozen, and no further adaptation was performed on any of the five evaluation databases.

The deliberate choice to train all ML and DL models exclusively on the CPSC dataset and evaluate on five fully disjoint external databases constitutes a strict cross-dataset generalization test without any risk of data leakage. This design was motivated by the practical deployment scenario in which no target-domain data is available for adaptation. However, we acknowledge that this setup does not constitute a cross-validation over training domains: it provides a lower bound on the performance attainable by data-driven approaches under idealized training conditions, rather than an estimate of their expected performance across the full range of possible deployment contexts. Readers should therefore interpret the ML/DL results as indicative of robustness under distribution shift from a single training source, not as a ceiling estimate of their achievable accuracy. A leave-one-dataset-out training scheme would provide a more balanced view of cross-domain performance, but was precluded by the scarcity of openly available and expert-annotated ECG datasets of sufficient size. We consider this a high-priority direction for future benchmarking.

### Selection of algorithms

The selection of algorithms followed a structured and criteria-driven approach to ensure a representative and meaningful benchmark. Four key criteria guided this process.

First, we aimed to achieve methodological diversity by including algorithms from different paradigms, namely traditional signal processing, classical ML, and DL approaches. This allows performance differences to be interpreted in light of underlying methodological characteristics rather than individual implementations. Second, we considered relevance and recency, focusing primarily on publications from the past decade (2013-2023) to reflect the current state of research, while also including widely used earlier approaches as reference methods. Third, reproducibility and transparency were prioritized. Algorithms were selected only if sufficiently detailed descriptions or source code were available to enable faithful re-implementation within a unified framework. Fourth, feasibility of consistent evaluation was ensured by including only algorithms that could be implemented and applied across all selected datasets under identical conditions. Reported performance in the original publications was not used as a selection criterion due to known inconsistencies in evaluation protocols across studies.

Based on these criteria, we conducted a structured literature search on PubMed using the query ((qrs[Title]) OR (r*peak[Title])) AND ((detect*[Title]) OR (locat*[Title])) for publications between 2013 and 2023, yielding 169 unique papers. Titles and abstracts were screened to identify the underlying methodological approaches, which were then categorized following a concept matrix-like procedure.

From these categories, representative algorithms were selected with the aim of covering the broadest possible range of technical approaches rather than maximizing reported performance. Table 2 provides an overview of the identified technical approaches and how they are represented by the selected algorithms.

This process resulted in a selection of 15 algorithms, including eight based on traditional signal processing and seven employing ML techniques (one clustering-based and six DL approaches). In addition, the well-established methods by Pan and Tompkins^1^ and Hamilton and Tompkins^34^ were included as reference algorithms. Full implementation details, including all concrete parameter values, are available in the accompanying GitHub repository (https://github.com/WIMUniCologne/rpeak_benchmarking).

**Table 2:** Overview of the technical approaches identified through the concept-based search, along with the corresponding selected algorithms. Some approaches are grouped under broader categories: for example, “Template-based” includes both template matching and template filtering, while “Deep Learning” encompasses 1D/2D convolutional neural networks (CNNs) and RNNs, all of which are represented in the selected algorithms.

	Basic signal processing	Digital filtering	Moving average	Peak envelope	Hilbert transformation	Shannon energy	Fourier transformation	Wavelet transformation	Empirical mode decomposition	Template-based	Machine learning	Deep learning
Pan^1^	$\blacksquare$	$\blacksquare$	$\blacksquare$									
Hamilton^34^	$\blacksquare$	$\blacksquare$	$\blacksquare$									
Elgendi^32^	$\blacksquare$	$\blacksquare$	$\blacksquare$									
Shaik^30^		$\blacksquare$					$\blacksquare$					
Park^42^	$\blacksquare$		$\blacksquare$	$\blacksquare$		$\blacksquare$		$\blacksquare$				
Arteaga-Falconi^43^	$\blacksquare$	$\blacksquare$										
Xu^28^	$\blacksquare$	$\blacksquare$		$\blacksquare$	$\blacksquare$	$\blacksquare$						
Nguyen^12^		$\blacksquare$								$\blacksquare$		
Zhai^31^	$\blacksquare$	$\blacksquare$								$\blacksquare$		
Kumari^35^	$\blacksquare$		$\blacksquare$	$\blacksquare$		$\blacksquare$			$\blacksquare$			
Xia^25^	$\blacksquare$							$\blacksquare$			$\blacksquare$	
Zahid^37^		$\blacksquare$									$\blacksquare$	$\blacksquare$
Laitala^39^											$\blacksquare$	$\blacksquare$
Han CNN^40^		$\blacksquare$						$\blacksquare$			$\blacksquare$	$\blacksquare$
Han RNN^40^		$\blacksquare$						$\blacksquare$			$\blacksquare$	$\blacksquare$
Xiang^26^		$\blacksquare$	$\blacksquare$								$\blacksquare$	$\blacksquare$
Çelik^38^								$\blacksquare$			$\blacksquare$	$\blacksquare$

## Results

### Metric definition

The definition of a true positive (TP), meaning a correctly detected R-peak, is inconsistent in the literature. R-peaks are often manually annotated by medical experts in ECG databases, but algorithms usually detect local maxima, which means they rarely match the exact point marked by the expert^31^. Automatically adjusting the annotations would therefore be problematic, especially since it could lead to errors with negative R-peaks. Additionally, filtering processes can slightly shift the signal, causing detected R-peaks to deviate from the annotations and be incorrectly classified as false positives (FPs) or false negatives (FNs)^1^. To compensate for such deviations, the literature suggests using a tolerance value ranging from 75^37^, 100^13^, to 150^31^ milliseconds. According to the IEC60601-2-47 standard for medical equipment, an R-peak is considered correctly detected if it lies within 75 milliseconds of the annotation^44^. Based on this standard, we evaluate the TPs.

We evaluate and assess the approaches using the following performance metrics, consistent with those commonly employed in the literature^11^:

$$\text {Recall} = \frac{TP}{TP + FN} \quad \text {Precision} = \frac{TP}{TP + FP} \quad \text {F1-Score} = 2 \cdot \frac{Precision \cdot Recall}{Precision + Recall} \quad \text {Error Rate} = \frac{FP + FN}{TP + FN}$$

Recall measures the proportion of correctly detected R-peaks among all true R-peaks, while precision quantifies the proportion of correct detections among all detected peaks. The F1-Score combines recall and precision, representing their harmonic mean. The error rate measures the ratio of falsely detected or missed R-peaks to the total number of R-peaks. Accuracy is not considered, as it can be misleading in highly imbalanced settings such as R-peak detection. For example, an algorithm that detects no R-peaks in a 10-second ECG segment could still achieve 99.8% accuracy due to the large number of true negatives (TNs).

Precision and recall are among the most commonly reported performance metrics in the literature, often complemented by error rate measures^11^. However, as our primary evaluation metric, we use the F1-Score, which provides a balanced assessment by combining precision and recall into a single measure.

To quantify statistical uncertainty, 95% confidence intervals for the F1-Score were estimated using bootstrap resampling at the recording level. Specifically, recordings were sampled with replacement over 10000 iterations, and the F1-Score was recomputed for each iteration. The 2.5th and 97.5th percentiles were used to derive the confidence intervals.

### Performance comparison

In this section, we present a comparative analysis of the evaluated algorithms across diverse datasets and conditions, highlighting their respective strengths and limitations rather than establishing a definitive ranking. The following interpretations should be considered in light of the chosen experimental setup, which evaluates cross-dataset generalization under a fixed, single-source training strategy without dataset-specific adaptation.

#### Performance per database

The best results in the MIT Long Term Database (Table 3) are achieved by the LSTM-network of Laitala et al.^39^, closely followed by Elgendi’s Blocks-of-Interest approach^32^ (after rounding, both achieve the same F1-Score, with the LSTM network achieving a lower error rate). Notably, the number of FPs and FNs of these two approaches is almost reversed, suggesting that combining both algorithms might help balance their individual weaknesses. Other algorithms perform significantly worse, with error rates around 5%, making them unusable in their current state.

**Table 3:** Detection performance metrics on the MIT Long Term Database. Bold values indicate the top-performing results for key metrics.

Total beats: 668735 Total duration: 147:27:27	Worst case				Sum over all recordings					
	Record no.	FP	FN	F1-Score	FP	FN	Recall	Precision	Error rate	F1-Score (95% CI)
Pan^1^	14046	54	6308	0.9716	390	12841	0.9808	0.9994	0.0198	0.9900 [0.9811–0.9978]
Hamilton^34^	14184	349	2313	0.9868	1529	6042	0.9910	0.9977	0.0113	0.9943 [0.9907–0.9976]
Elgendi^32^	14172	240	1	0.9982	432	**247**	**0.9996**	0.9994	**0.0010**	**0.9995 [0.9991–0.9998]**
Shaik^30^	14046	39	9685	0.9560	3282	25018	0.9626	0.9949	0.0423	0.9785 [0.9691–0.9890]
Park^42^	14184	0	31669	0.8153	258	51601	0.9228	0.9996	0.0775	0.9597 [0.9001–0.9964]
Arteaga-Falconi^43^	14046	401	1353	0.9924	989	5036	0.9925	0.9985	0.0090	0.9955 [0.9939–0.9976]
Xu^28^	14046	62	9412	0.9572	294	15684	0.9765	0.9995	0.0239	0.9879 [0.9759–0.9971]
Nguyen^12^	14046	192	5558	0.9745	1141	12349	0.9815	0.9983	0.0202	0.9898 [0.9813–0.9975]
Zhai^31^	14172	579	575	0.9913	1250	1361	0.9980	0.9981	0.0039	0.9980 [0.9961–0.9992]
Kumari^35^	14046	182	27063	0.8662	8109	28609	0.9572	0.9875	0.0549	0.9721 [0.9300–0.9944]
Xia^25^	14184	0	2621	0.9869	427	5983	0.9911	0.9994	0.0096	0.9952 [0.9912–0.9985]
Zahid^37^	15814	3094	2309	0.9740	6017	3874	0.9942	0.9910	0.0148	0.9926 [0.9852–0.9986]
Laitala^39^	14046	65	189	0.9989	**220**	420	0.9994	**0.9997**	**0.0010**	**0.9995 [0.9992–0.9998]**
Han CNN^40^	14149	4116	3936	0.9722	5897	5672	0.9915	0.9912	0.0173	0.9913 [0.9826–0.9980]
Han RNN^40^	14149	4884	1354	0.9787	6326	4395	0.9934	0.9906	0.0160	0.9920 [0.9854–0.9984]
Xiang^26^	14184	4163	4	0.9799	10285	609	0.9991	0.9848	0.0163	0.9919 [0.9864–0.9959]
Çelik^38^	14046	15187	8100	0.9020	17707	13916	0.9792	0.9737	0.0473	0.9764 [0.9456–0.9966]

**Table 4:** Detection performance metrics on the Fantasia Database. Bold values indicate the top-performing results for key metrics.

Total beats: 282583 Total duration: 79:46:23	Worst case				Sum over all recordings					
	Record no.	FP	FN	F1-Score	FP	FN	Recall	Precision	Error rate	F1-Score (95% CI)
Pan^1^	f2o08	850	5	0.9372	2741	35	**0.9999**	0.9904	**0.0098**	**0.9951 [0.9917–0.9972]**
Hamilton^34^	f2o05	92	8050	0.0725	2700	8258	0.9708	0.9903	0.0388	0.9804 [0.9487–0.9969]
Elgendi^32^	f2o08	852	6	0.9370	3372	59	0.9998	0.9882	0.0121	0.9940 [0.9901–0.9967]
Shaik^30^	f2o08	821	64	0.9346	3076	2729	0.9903	0.9891	0.0205	0.9897 [0.9853–0.9935]
Park^42^	f2o08	842	8	0.9376	2606	177	0.9994	**0.9909**	**0.0098**	**0.9951 [0.9917–0.9972]**
Arteaga-Falconi^43^	f2o08	850	9	0.9369	3396	103	0.9996	0.9881	0.0124	0.9938 [0.9901–0.9965]
Xu^28^	f2o08	840	13	0.9373	**2631**	676	0.9976	0.9908	0.0117	0.9942 [0.9904–0.9969]
Nguyen^12^	f2o08	852	5	0.9371	3037	36	**0.9999**	0.9894	0.0109	0.9946 [0.9912–0.9968]
Zhai^31^	f2o08	852	4	0.9372	3410	**34**	**0.9999**	0.9881	0.0122	0.9939 [0.9902–0.9966]
Kumari^35^	f1y05	1	6950	0.0000	20203	33608	0.8811	0.9249	0.1904	0.9025 [0.8365–0.9509]
Xia^25^	f2o08	850	5	0.9372	3174	38	**0.9999**	0.9889	0.0114	0.9943 [0.9905–0.9969]
Zahid^37^	f2o08	844	6	0.9376	3037	123	0.9996	0.9894	0.0112	0.9944 [0.9907–0.9971]
Laitala^39^	f2o08	845	5	0.9376	3128	46	0.9998	0.9891	0.0112	0.9944 [0.9907–0.9971]
Han CNN^40^	f2o08	844	7	0.9375	3496	68	0.9998	0.9878	0.0126	0.9937 [0.9900–0.9964]
Han RNN^40^	f2o08	842	6	0.9377	4280	102	0.9996	0.9851	0.0155	0.9923 [0.9888–0.9949]
Xiang^26^	f1o09	3822	1	0.7163	8367	803	0.9972	0.9712	0.0325	0.9840 [0.9683–0.9936]
Çelik^38^	f2o08	844	7	0.9375	3454	56	0.9998	0.9879	0.0124	0.9938 [0.9900–0.9968]

In the Fantasia Database (Table 4), 13 out of 17 algorithms achieve F1-Scores above 0.99. The main reason for that is probably that the recordings in this database were taken from relaxed patients, leading to less movement and therefore less noise. The algorithms by Pan and Tompkins^1^, as well as Park et al.^42^, achieve the best detection results with identical error rates and F1-Scores. Interestingly, the algorithm by Park et al.^42^, which was among the worst performers in the MIT Long Term Database, ranks among the best here, indicating data-dependent fluctuations in detection performance. The results of the Pan and Tompkins algorithm^1^ further demonstrate that, despite its age, it can outperform modern algorithms in specific scenarios.

**Table 5:** Detection performance metrics on the Pulse Transit Time Database. Bold values indicate the top-performing results for key metrics.

Total beats: 46405 Total duration: 9:00:34	Worst case				Sum over all recordings					
	Record no.	FP	FN	F1-Score	FP	FN	Recall	Precision	Error rate	F1-Score (95% CI)
Pan^1^	s12_run	42	30	0.9510	147	65	0.9986	0.9968	0.0046	0.9977 [0.9955–0.9994]
Hamilton^34^	s12_run	36	27	0.9570	167	62	0.9987	0.9964	0.0049	0.9975 [0.9958–0.9989]
Elgendi^32^	s12_run	40	5	0.9698	161	36	0.9992	0.9965	0.0042	0.9979 [0.9963–0.9992]
Shaik^30^	s12_walk	51	2	0.9639	401	61	0.9987	0.9914	0.0100	0.9950 [0.9926–0.9971]
Park^42^	s13_sit	21	16	0.9694	59	111	0.9976	**0.9987**	**0.0037**	**0.9982 [0.9967–0.9994]**
Arteaga-Falconi^43^	s12_run	21	43	0.9554	96	110	0.9976	0.9979	0.0044	0.9978 [0.9959–0.9992]
Xu^28^	s12_run	34	46	0.9446	134	123	0.9973	0.9971	0.0055	0.9972 [0.9949–0.9990]
Nguyen^12^	s12_run	37	33	0.9521	122	74	0.9984	0.9974	0.0042	0.9979 [0.9957–0.9995]
Zhai^31^	s12_run	96	19	0.9250	507	38	0.9992	0.9892	0.0117	0.9942 [0.9901–0.9974]
Kumari^35^	s12_walk	86	0	0.9429	557	27	0.9994	0.9881	0.0126	0.9937 [0.9906–0.9963]
Xia^25^	s12_run	78	17	0.9374	282	33	0.9993	0.9940	0.0068	0.9966 [0.9936–0.9989]
Zahid^37^	s12_run	21	38	0.9590	**56**	123	0.9973	0.9988	0.0039	0.9981 [0.9963–0.9994]
Laitala^39^	s12_run	75	12	0.9427	512	27	0.9994	0.9891	0.0116	0.9942 [0.9911–0.9969]
Han CNN^40^	s2_run	12	108	0.9455	214	228	0.9951	0.9954	0.0095	0.9952 [0.9919–0.9978]
Han RNN^40^	s12_run	14	19	0.9773	157	67	0.9986	0.9966	0.0048	0.9976 [0.9965–0.9985]
Xiang^26^	s12_run	63	9	0.9523	365	158	0.9966	0.9822	0.0113	0.9944 [0.9919–0.9965]
Çelik^38^	s12_walk	184	0	0.8853	1085	**15**	**0.9997**	0.9771	0.0237	0.9883 [0.9819–0.9937]

**Table 6:** Detection performance metrics on the MIT Arrhythmia Database.

Total beats: 109494 Total duration: 24:04:27	Worst case				Sum over all recordings					
	Record no.	FP	FN	F1-Score	FP	FN	Recall	Precision	Error rate	F1-Score (95% CI)
Pan^1^	207	182	55	0.9384	462	1611	0.9853	0.9957	0.0189	0.9905 [0.9860–0.9945]
Hamilton^34^	207	283	40	0.9185	877	1346	0.9877	0.9920	0.0203	0.9898 [0.9845–0.9942]
Elgendi^32^	207	306	8	0.9219	553	179	0.9984	0.9950	**0.0067**	**0.9967 [0.9933–0.9988]**
Shaik^30^	207	287	2	0.9278	672	381	0.9965	0.9939	0.0096	0.9952 [0.9916–0.9979]
Park^42^	208	0	900	0.8204	318	2688	0.9755	0.9970	0.0275	0.9861 [0.9762–0.9938]
Arteaga-Falconi^43^	203	94	362	0.9199	702	1452	0.9867	0.9935	0.0197	0.9901 [0.9846–0.9946]
Xu^28^	203	11	489	0.9087	**174**	1848	0.9831	**0.9984**	0.0185	0.9907 [0.9846–0.9954]
Nguyen^12^	208	2	589	0.8890	340	2058	0.9812	0.9968	0.0219	0.9890 [0.9822–0.9944]
Zhai^31^	207	156	37	0.9497	369	805	0.9926	0.9966	0.0107	0.9946 [0.9911–0.9974]
Kumari^35^	207	56	260	0.9101	712	690	0.9937	0.9935	0.0128	0.9936 [0.9895–0.9969]
Xia^25^	207	145	61	0.9458	442	753	0.9931	0.9960	0.0109	0.9945 [0.9915–0.9971]
Zahid^37^	208	4	489	0.9091	363	1319	0.9880	0.9967	0.0154	0.9923 [0.9865–0.9967]
Laitala^39^	113	160	0	0.9573	419	378	0.9965	0.9962	0.0073	0.9964 [0.9939–0.9983]
Han CNN^40^	207	284	9	0.9267	592	1063	0.9903	0.9946	0.0151	0.9924 [0.9875–0.9963]
Han RNN^40^	207	267	13	0.9295	526	1032	0.9906	0.9952	0.0142	0.9929 [0.9888–0.9963]
Xiang^26^	231	211	1	0.9368	442	493	0.9955	0.9960	0.0085	0.9957 [0.9929–0.9980]
Çelik^38^	207	653	1	0.8504	1868	**99**	**0.9991**	0.9832	0.0180	0.9911 [0.9832–0.9966]

For the Pulse Transit Time Database (Table 5), with the exception of the algorithm by Xiang et al.^26^, all algorithms achieve average F1-Scores well above 0.99, with nine of them even reaching values above 0.997. The low number of detection errors suggests that the differences between these nine algorithms are hardly significant. Both the algorithms by Pan and Tompkins^1^ and Hamilton and Tompkins^34^ achieve results close to the best, highlighting their continued relevance. Additionally, the algorithm by Park et al.^42^ demonstrates its data-dependent performance, achieving the highest F1-Score for this specific database, despite its relatively lower performance on the MIT Long Term Database. This suggests that while Park et al.^42^ may be particularly well-suited to certain databases, its generalizability remains limited.

Most algorithms show low recall to arrhythmic beats regarding the MIT Arrhythmia Database (Table 6). Specifically, the algorithms by Pan and Tompkins^1^ as well as Hamilton and Tompkins^34^ perform poorly, which is attributed to their use of correction techniques that rely on the regular rhythm of R-peaks, a pattern disrupted in the MIT Arrhythmia Database. Interestingly, the algorithm by Park et al.^42^ achieves the worst results in this database, despite being among the best performers in the Pulse Transit Time and Fantasia Databases. In contrast, the algorithms by Çelik et al.^38^ and Xiang et al.^26^ perform well here, even though they had weaker performances in the MIT Long Term Database.

**Table 7:** Detection performance metrics on the MIT Noise Stress Test Database. Bold values indicate the top-performing results for key metrics.

Total beats: 25590 Total duration: 6:01:07	Worst case				Sum over all recordings					
	Record no.	FP	FN	F1-Score	FP	FN	Recall	Precision	Error rate	F1-Score (95% CI)
Pan^1^	118e_6	645	1197	0.5400	3013	1756	0.9314	0.8878	0.1864	0.9091 [0.8308–0.9691]
Hamilton^34^	118e_6	659	471	0.7618	3419	1342	0.9476	0.8764	0.1860	0.9106 [0.8641–0.9536]
Elgendi^32^	119e_6	819	183	0.7826	3892	644	0.9748	0.8650	0.1773	0.9167 [0.8720–0.9603]
Shaik^30^	119e_6	628	227	0.8046	2732	709	0.9723	0.9011	0.1345	0.9353 [0.8959–0.9710]
Park^42^	119e_6	431	537	0.7497	1580	2320	0.9093	**0.9364**	0.1523	0.9227 [0.8740–0.9645]
Arteaga-Falconi^43^	118e_6	696	710	0.6904	3221	1759	0.9313	0.8809	0.1946	0.9054 [0.8422–0.9606]
Xu^28^	118e_6	333	1294	0.5474	1662	3413	0.8666	0.9303	0.1983	0.8973 [0.8081–0.9667]
Nguyen^12^	118e_6	609	1411	0.4619	2707	2250	0.9121	0.8961	0.1937	0.9040 [0.8166–0.9684]
Zhai^31^	118e_6	642	1495	0.4229	3740	2263	0.9116	0.8618	0.2346	0.8860 [0.7920–0.9563]
Kumari^35^	119e00	107	1873	0.1033	**1249**	10218	0.6007	0.9249	0.4481	0.7283 [0.4948–0.8949]
Xia^25^	119e_6	800	255	0.7665	3853	496	0.9806	0.8669	0.1699	0.9203 [0.8756–0.9629]
Zahid^37^	119e_6	478	373	0.7914	2121	1902	0.9257	0.9178	0.1572	0.9217 [0.8823–0.9590]
Laitala^39^	119e_6	628	535	0.7140	3430	1542	0.9397	0.8752	0.1943	0.9063 [0.8524–0.9547]
Han CNN^40^	119e_6	571	204	0.8215	2579	665	0.9740	0.9062	0.1268	0.9389 [0.9026–0.9720]
Han RNN^40^	119e_6	587	198	0.8201	2490	569	0.9778	0.9095	**0.1195**	**0.9424 [0.9056–0.9753]**
Xiang^26^	119e_6	558	254	0.8102	2749	990	0.9614	0.8997	0.1459	0.9295 [0.8899–0.9675]
Çelik^38^	119e_6	828	149	0.7900	5194	**323**	**0.9874**	0.8295	0.2156	0.9016 [0.8625–0.9409]

**Table 8:** Peak-wise detection performance metrics on all five evaluation databases, sorted descending by F1-Score. Bold values indicate the top-performing results for key metrics.

Total beats: 1132807	FP	FN	Recall	Precision	Error rate	F1-Score (95% CI)
Elgendi^32^	8410	**1165**	**0.9990**	0.9926	**0.0085**	**0.9958 [0.9922–0.9942]**
Laitala^39^	7709	2413	0.9979	0.9932	0.0089	0.9955 [0.9919–0.9975]
Zhai^31^	9276	4501	0.9960	0.9918	0.0122	0.9939 [0.9895–0.9965]
Xia^25^	8178	7303	0.9936	0.9928	0.0137	0.9932 [0.9898–0.9957]
Arteaga-Falconi^43^	8404	8460	0.9925	0.9926	0.0149	0.9926 [0.9891–0.9947]
Zahid^37^	11594	7341	0.9935	0.9898	0.0167	0.9917 [0.9869–0.9955]
Han RNN^40^	13779	6165	0.9946	0.9879	0.0176	0.9912 [0.9869–0.9951]
Han CNN^40^	12778	7696	0.9932	0.9888	0.0181	0.9910 [0.9852–0.9953]
Pan^1^	6753	16308	0.9856	0.9940	0.0204	0.9898 [0.9840–0.9948]
Nguyen^12^	7347	16767	0.9852	0.9935	0.0213	0.9893 [0.9895–0.9942]
Xiang^26^	22202	3053	0.9973	0.9807	0.0223	0.9889 [0.9828–0.9930]
Hamilton^34^	8692	17050	0.9849	0.9923	0.0227	0.9886 [0.9777–0.9942]
Xu^28^	4895	21744	0.9808	**0.9956**	0.0235	0.9882 [0.9806–0.9940]
Shaik^30^	10163	28898	0.9745	0.9909	0.0345	0.9826 [0.9765–0.9895]
Çelik^38^	29308	14409	0.9873	0.9745	0.0386	0.9808 [0.9618–0.9930]
Park^42^	**4821**	56897	0.9498	0.9955	0.0545	0.9721 [0.9402–0.9935]
Kumari^35^	30830	73152	0.9354	0.9717	0.0918	0.9532 [0.9214–0.9754]

**Table 9:** Average detection performance across the five benchmark databases. Values denote the arithmetic mean of recall, precision, error rate, and F1-Score over all databases. Entries are sorted descending by F1-Score. Bold values indicate the top-performing results for key metrics.

	Avg. recall	Avg. precision	Avg. error rate	Avg. F1-Score
Han RNN^40^	0.9920	0.9754	**0.0340**	**0.9834**
Han CNN^40^	0.9901	0.9750	0.0363	0.9823
Elgendi^32^	**0.9944**	0.9688	0.0403	0.9810
Xia^25^	0.9928	0.9690	0.0417	0.9802
Zahid^37^	0.9810	0.9787	0.0405	0.9798
Xiang^26^	0.9900	0.9668	0.0429	0.9791
Shaik^30^	0.9841	0.9741	0.0434	0.9787
Laitala^39^	0.9870	0.9699	0.0451	0.9782
Arteaga-Falconi^43^	0.9815	0.9718	0.0480	0.9765
Pan^1^	0.9792	0.9740	0.0479	0.9765
Nguyen^12^	0.9746	0.9756	0.0502	0.9751
Hamilton^34^	0.9792	0.9706	0.0523	0.9745
Xu^28^	0.9642	0.9832	0.0516	0.9735
Zhai^31^	0.9803	0.9668	0.0546	0.9733
Park^42^	0.9609	**0.9845**	0.0542	0.9724
Çelik^38^	0.9930	0.9503	0.0634	0.9702
Kumari^35^	0.8864	0.9638	0.1438	0.9180

The MIT Noise Stress Test Database was specifically designed to challenge algorithms with highly noisy recordings. Consequently, the lower performance metrics observed across all algorithms are expected, with their importance lying more in relative comparisons than in absolute values (Table 7). The RNN and CNN by Han et al.^40^ achieve the highest F1-Scores for both the worst-case recording and all recordings combined, making them the most noise-robust algorithms according to this database. However, a universal application of these algorithms might be shortsighted, as they did not always deliver the best results in other databases.

#### Performance overall

The overall results were calculated by summing all R-peak annotations and detection errors (FP and FN) across the five evaluation databases (Table 8). This beat-wise aggregation assigns equal weight to each individual R-peak and serves as our primary ranking criterion. We consider this appropriate because the included databases differ substantially in size, recording purpose, and difficulty. Under a simple arithmetic mean across databases, small but highly specific datasets would contribute as much to the final ranking as substantially larger ones, for instance, giving the 6-hour MIT Noise Stress Test Database, which contains deliberately noise-corrupted recordings, equal influence to the 147-hour MIT Long Term Database. This would overemphasize edge-case conditions and could distort conclusions about expected out-of-the-box performance in broader practical use. From an application-oriented perspective, the beat-wise aggregation is more closely aligned with the actual deployment setting, in which algorithms process individual beats rather than datasets, and each falsely detected or missed R-peak matters irrespective of the database from which it originates.

Nevertheless, we additionally report the arithmetic mean across databases as a complementary perspective (Table 9), as it captures how consistently an algorithm performs when each benchmark domain is treated as equally important, regardless of size. This perspective may be particularly relevant for readers interested in whether an algorithm remains competitive across fundamentally different recording environments, including arrhythmic, long-term, and strongly noise-contaminated settings. The two rankings should therefore be interpreted as reflecting complementary viewpoints rather than a single correct ordering.

The resulting beat-weighted performance ranking, based on F1-Score, identified Elgendi’s Blocks-of-Interest method^32^ as the best-performing overall approach, followed closely by Laitala et al.’s LSTM-network^39^ and Zhai et al.’s template matching technique^31^. All three algorithms tend to produce more FPs than FNs. The following top five best-performing algorithms, most using ML, also showed strong results with F1-Scores above 0.9900. These outcomes were reached without any data-specific training or parameter optimization, suggesting that the top-performing algorithms are relatively robust to variations in input data. However, this might also explain the underperformance of other algorithms, like Park et al.^42^, which, despite excelling on smaller databases, showed poor results on the larger MIT Long Term Database, leading to the second-worst overall performance. Han et al.’s RNN^40^ achieved the best results on the MIT Noise Stress Test Database but performed only moderately on others.

**Fig. 4:**
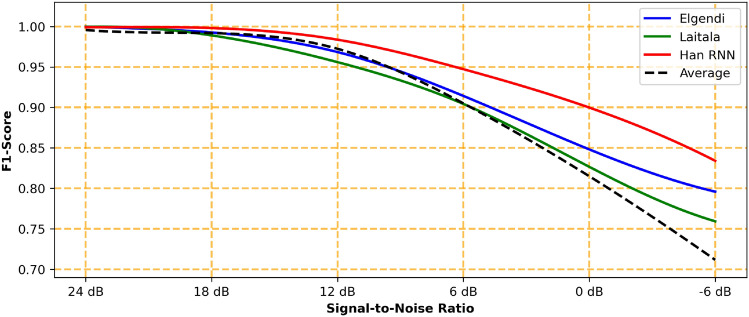
Average peak-wise detection performance achieved by the algorithms under varying levels of signal-to-noise ratio (SNR) based on the recordings from the MIT Noise Stress Test Database: The RNN by Han et al.^40^ exhibited the highest noise robustness. Under high levels of noise (lower SNR), its detection performance is significantly higher than the one achieved by the algorithms of Elgendi^32^ and Laitala et al.^39^. For better readability, the detection performance of the remaining algorithms is represented by the black dashed line as their average F1-Score.

**Table 10:** Development of the detection performance as the degree of movement increases. Bold values indicate the top-performing results for key metrics.

	F1-Score			Performance difference (%)	
	Sit	Walk	Run	Sit vs. Walk	Sit vs. Run
Pan^1^	0.9987	0.9973	0.9973	− 0.14	− 0.14
Hamilton^34^	0.9980	0.9976	0.9970	− 0.04	− 0.10
Elgendi^32^	0.9983	0.9975	**0.9977**	− 0.08	− 0.06
Shaik^30^	0.9984	0.9949	0.9920	− 0.35	− 0.64
Park^42^	0.9984	0.9985	0.9979	0.01	− 0.05
Arteaga-Falconi^43^	0.9985	0.9979	0.9971	− 0.06	− 0.14
Xu^28^	0.9983	0.9972	0.9963	− 0.11	− 0.20
Nguyen^12^	0.9986	0.9977	0.9973	− 0.09	− 0.13
Zhai^31^	0.9985	0.9932	0.9915	− 0.53	− 0.70
Kumari^35^	0.9961	0.9927	0.9922	− 0.34	− 0.39
Xia^25^	0.9986	0.9957	0.9959	− 0.29	− 0.27
Zahid^37^	**0.9990**	**0.9988**	0.9968	− 0.02	− 0.22
Laitala^39^	0.9986	0.9937	0.9906	− 0.49	− 0.80
Han CNN^40^	0.9978	0.9960	0.9938	− 0.18	− 0.40
Han RNN^40^	0.9973	0.9982	0.9972	0.09	− 0.01
Xiang^26^	0.9963	0.9946	0.9921	− 0.17	− 0.42
Çelik^38^	0.9966	0.9863	0.9830	− 1.03	− 1.36

#### Influence of noise

Many algorithms perform well with high-quality ECG recordings, but their detection accuracy decreases as noise levels increase (Fig. 4). This pattern is evident in the *Performance per Database* Chapter, where Elgendi’s^32^ and Laitala et al.’s^39^ algorithms, despite strong overall performance, showed only moderate results on the MIT Noise Stress Test Database. Zhai et al.’s^31^ algorithm, which ranked third overall, performed even worse in noisy conditions.

Conversely, Han et al.’s RNN^40^ was more robust to noise, performing worse with high-quality signals. This suggests that relying solely on the best overall or the most noise-resistant algorithms is not ideal when both weak and strong noise levels need to be considered, highlighting the need for a noise-dependent algorithm selection strategy.

Another factor affecting detection performance, especially in everyday situations, is noise caused by motion artifacts. These can be observed in the Pulse Transit Time Database, which contains recordings taken while sitting, walking, and running. Comparing the differences between sitting and walking, as well as sitting and running, shows that motion artifacts have a measurable impact. Although the performance decrease is small (Table 10), it is noticeable and consistent: 15 out of 17 algorithms showed reduced performance when patients were walking, and all 17 performed worse when patients were running compared to when they were sitting. Even slight reductions in performance can have significant consequences in sensitive applications like seizure detection, where a single missed or incorrectly detected R-Peak can affect the outcome^9^.

Notably, Han et al.‘s RNN^40^ demonstrated the most stable performance across these conditions, even though it did not achieve the best overall results. This aligns with its earlier assessment, where it was noted for its high noise robustness, despite not being the top performer overall.

## Discussion

The central question motivating this study is which R-peak detection algorithms generalize well when applied out of the box, without dataset-specific optimization or prior knowledge of patient characteristics. To investigate this, we benchmarked a broad and representative selection of algorithms from different methodological paradigms. Given practical limitations, not every existing approach could be included; our aim was to represent broader algorithmic classes rather than evaluate every specific implementation. In most applications, the specific characteristics of ECGs are not known in advance, making data-specific optimization unfeasible. Therefore, an approach that performs robustly across patient and domain heterogeneity is required. Under these conditions, Elgendi’s Blocks-of-Interest algorithm^32^, followed by Laitala et al.’s LSTM^39^, showed the best overall beat-wise performance, providing balanced performance across datasets. Performance could be further enhanced by switching to Han et al.’s RNN^40^ when high noise levels are expected, such as during movement-based data collection.

These performance patterns can be partly explained by the design assumptions underlying each approach. Traditional signal processing methods such as Elgendi’s algorithm^32^ rely on handcrafted rules derived from known physiological properties of the QRS complex, such as its characteristic amplitude and duration. This makes them relatively robust to distribution shifts, as these fundamental signal properties tend to remain stable across patients and recording conditions. In contrast, DL models learn their feature representations from training data, which can enable strong performance when training and evaluation data share similar characteristics, but may lead to degraded performance when faced with unfamiliar noise patterns or ECG morphologies. The noise robustness of Han et al.’s RNN^40^, for instance, may be attributed to the ability of recurrent architectures to leverage temporal context across beats, effectively smoothing over localized noise. Conversely, algorithms that rely on regular RR-interval timing, such as Pan and Tompkins^1^, can struggle with arrhythmic recordings where this assumption is violated.

The per-database results also allow some systematic observations regarding recurring failure patterns, such as elevated false negative rates for rhythm-dependent methods on arrhythmic data, performance degradation of threshold-based approaches under high noise, and limited generalization of DL models trained on narrow distributions (see also Tables 3, 6, 7). However, a comprehensive, structured taxonomy of failure modes is beyond the scope of this benchmarking study. A dedicated investigation that systematically maps algorithmic design assumptions to specific signal characteristics and failure conditions at the level of individual segments and beat morphologies would be a valuable direction for future research.

Importantly, our results should be interpreted in the context of the adopted training strategy: all ML and DL models were trained exclusively on the CPSC dataset and evaluated on unseen external databases without any form of domain adaptation or dataset-specific fine-tuning. While this design enables a strict assessment of cross-dataset robustness and avoids data leakage, it may also lead to an underestimation of the achievable performance of data-driven approaches. Ideally, training would be conducted on multiple diverse datasets and evaluation on a separate, equally diverse set of databases. However, the limited availability of openly accessible, expert-annotated ECG databases of sufficient quality and size imposes practical constraints on this approach. Training on a more diverse range of datasets, as demonstrated by Yun et al.^45^, has the potential to improve algorithmic performance, and we consider expanding the training basis a valuable direction for future work. Accordingly, the comparative results presented here primarily reflect robustness under distribution shifts rather than maximum attainable performance under ideal training conditions. When dataset-specific optimization is feasible, for instance in retrospective studies with well-defined datasets, substantially improved results may be expected, though at the cost of reduced generalizability and with the need for annotated data.

This persistent trade-off between peak performance and generalizability likely explains why R-peak detection remains an active field of research, and why the Pan-Tompkins algorithm^1^, despite being almost 40 years old, continues to deliver competitive results. Given that the top-performing algorithms produced complementary error patterns, with some methods favoring precision and others recall, ensemble strategies that combine multiple detectors could be a promising direction for future work. Such approaches might leverage the individual strengths observed in this benchmark, for instance combining Elgendi’s consistent overall performance with Han et al.’s noise robustness, to further reduce both false positives and false negatives. This is particularly relevant since even a single incorrectly detected peak can lead to significant problems in downstream analyses^9^.

Although our study provides a comprehensive benchmark across multiple datasets, several limitations should be acknowledged. A key limitation of this study lies in the training strategy for ML/DL models, which relies on a single dataset. While this enables a strict evaluation of cross-dataset generalization, it does not reflect common practice where models are trained on multiple diverse datasets or fine-tuned to the target domain. As a result, the reported performance of ML/DL approaches may underestimate their achievable performance under more favorable training conditions. Furthermore, the five PhysioNet datasets may not fully represent all real-world ECG variations; scenarios involving multi-lead clinical recordings, intensive care unit monitoring, or wearable devices with strong motion artifacts are only partially captured. The conclusions derived from this study therefore predominantly reflect performance under the specific conditions of the considered datasets and may not be entirely applicable to all real-world scenarios. In addition, while we re-implemented several algorithms based on published descriptions, differences in parameter tuning and implementation details could introduce unintended performance discrepancies. Finally, we intentionally did not focus on memory and runtime consumption. Nevertheless, these factors are crucial for real-world applications: algorithms like those by Nguyen et al.^12^ are optimized for minimal resource use, while computationally intensive DL approaches like Han’s RNN^40^ require significant computing resources. Future work should investigate additional datasets, real-world noise conditions, algorithm memory and runtime consumption, as well as hardware constraints to further refine algorithm selection for diverse clinical and practical applications.

## Conclusion

Our findings suggest that a single, universally optimal algorithm for R-peak detection does not exist. Instead, it is crucial to evaluate algorithms under varying conditions and across diverse datasets to transparently reveal their strengths and weaknesses, enabling informed decisions tailored to specific applications and data characteristics. Under the cross-dataset generalization scenario evaluated here, signal processing-based methods delivered more consistent results, while ML and DL techniques exhibited stronger data-dependency. These findings highlight the importance of training distribution breadth for data-driven approaches rather than a fundamental limitation of the paradigm itself. Conclusions about the relative merits of each approach should therefore be contextualized by the constraints of the adopted training strategy. We encourage the community to adopt and extend the open evaluation framework presented in this study, including the datasets and metrics defined here, to facilitate continued transparent and comparable benchmarking of R-peak detection algorithms.

## Data Availability

The ECGs used in this study were from open source databases and available in https://physionet.org/about/database/ (MIT Long Term, Fantasia, Pulse Transit Time, MIT Arrhythmia, and MIT Noise Stress Test databases) and http://icbeb2020.pastconf.com/CSPC2020 (CPSC database). The complete benchmarking framework, including all algorithm implementations, preprocessing steps, and evaluation procedures, is publicly available at: https://github.com/WIMUniCologne/rpeak_benchmarking. This ensures full reproducibility of all reported results and facilitates reuse and extension by future research.
